# Association Between Neutrophil Percentage‐to‐Albumin Ratio (NPAR) and Risk of Stroke in Patients With Hypertension: A Cohort Study

**DOI:** 10.1111/jch.70200

**Published:** 2026-01-16

**Authors:** Leyi Wang, Zhihao Liu, Nan Zhang, Long Zhang, Xu Liu, Fangfang Fan, Yan Zhang, Jianping Li

**Affiliations:** ^1^ Department of Cardiology Peking University First Hospital Beijing China; ^2^ Institute of Cardiovascular Disease Peking University First Hospital Beijing China; ^3^ State Key Laboratory of Vascular Homeostasis and Remodeling Peking University Beijing China; ^4^ NHC Key Laboratory of Cardiovascular Molecular Biology and Regulatory Peptides Peking University Beijing China

**Keywords:** hypertension, inflammation, neutrophil percentage‐to‐albumin ratio, sex difference stroke

## Abstract

Stroke is a leading cause of disability among hypertensive adults, with notable sex differences in risk and outcomes. Neutrophil percentage‐to‐albumin ratio (NPAR) is an easily obtainable composite index of systemic inflammation with prognostic value in cardiovascular disease, but its utility for primary stroke prevention in hypertension remains unclear. We therefore examined the association of NPAR with first stroke in hypertensive adults and tested for sex‐specific effects. We analyzed 13 848 participants from the China Stroke Primary Prevention Trial. NPAR was calculated as the neutrophil percentage (%) × 100/albumin (g/dL). Cox proportional hazards models evaluated the association between NPAR and first stroke, and subgroup analyses assessed sex‐specific effects. During a median follow‐up of 4.5 years, 371 participants (2.7%) experienced stroke. The risk of stroke was significantly higher in Q2, Q3, and Q4 than in Q1 (HR 1.76, 95% CI 1.31–2.36, *p* < 0.001 [Q2], HR 1.54, 95% CI 1.14–2.08, *p* = 0.005 [Q3], and HR 1.57, 95% CI 1.17–2.12, *p* = 0.003 [Q4] in the adjusted model). The result remained consistent when the Q2 to Q4 groups were combined and compared with the Q1 group. Subgroup analysis revealed a significant sex difference, with higher NPAR associated with increased stroke risk in women but not in men (*p* = 0.035). These findings suggest that higher NPAR independently predicts stroke risk in patients with hypertension, with a substantially stronger association in women, and highlight sex‐specific inflammatory mechanisms and the potential of NPAR as a biomarker for female‐focused prevention strategies.

**Abbreviations**
BMI: body mass index
CRP: C‐reactive protein
CSPPT: China Stroke Primary Prevention Trial
CVD: cardiovascular disease
DBP: diastolic blood pressure
eGFR: estimated glomerular filtration rate
FBG: fasting blood glucose
HDL‐C: high‐density lipoprotein cholesterol
LDL‐C: low‐density lipoprotein cholesterol
NPAR: neutrophil percentage‐to‐albumin ratio
SBP: systolic blood pressure
TC: total cholesterol
TG: triglycerides

## 1 Background

Stroke remains one of the most significant global health challenges, accounting for the highest burden among neurological disorders and contributing to widespread mortality and disability [1, 2, 3].

A wide range of conventional risk factors, including hypertension and hyperlipidemia, are known to be associated with stroke. Although interventions targeting these risk factors have significantly diminished the incidence of stroke, there are still unidentified residual risks associated with stroke that these interventions do not fully address [4, 5, 6]. Further investigation of these residual risks is needed to overcome the current plateau in the incidence of stroke and maintain a downward trend.

Recent studies have identified inflammation to be a risk factor for stroke and a key driver of atherosclerotic plaque instability and subsequent thromboembolic events [7]. Among the emerging biomarkers, neutrophil percentage‐to‐albumin ratio (NPAR) has attracted attention as a simple and cost‐effective measure of systemic inflammation. The NPAR, calculated as the ratio of neutrophil percentage to serum albumin concentration, has demonstrated significant predictive value for adverse outcomes in a range of conditions, including heart failure [8, 9], acute myocardial infarction [10, 11, 12], and atrial fibrillation [13, 14], as well as in patients in cardiac intensive care units [15, 16, 17], those with acute kidney injury [18] or severe infection [19, 20], and those undergoing peritoneal dialysis [21, 22, 23]. Previous research on cerebrovascular disease has focused mainly on secondary prevention. NPAR has been shown to predict the recurrence of first‐episode ischemic stroke [24], stroke‐associated infection [25, 26, 27], and the functional and clinical prognosis of stroke [28, 29]. Nevertheless, there is limited literature on the value of the NPAR in the primary prevention of stroke, and its ability to predict the risk of stroke in patients with hypertension has yet to be explored.

This study sought to address this gap by prospectively investigating the association between NPAR and the risk of stroke in patients with hypertension.

## 2 Methods

### 2.1 Study Design and Population

All participants were recruited from the China Stroke Primary Prevention Trial (CSPPT). Detailed information about the CSPPT cohort has been published elsewhere [30]. In brief, the CSPPT was a multi‐community, randomized, double‐blind controlled trial that assessed the ability of folic acid therapy to reduce the risk of first stroke in Chinese patients with hypertension. The trial was conducted across 32 communities in China between May 19, 2008, and August 24, 2013, and included 20 702 patients aged 45–75 years with hypertension. During a median follow‐up of 4.5 years, a total of 637 stroke events were recorded. The results demonstrated that the combination of enalapril and folic acid was more effective in reducing the risk of first stroke than enalapril alone.

**FIGURE 1:**
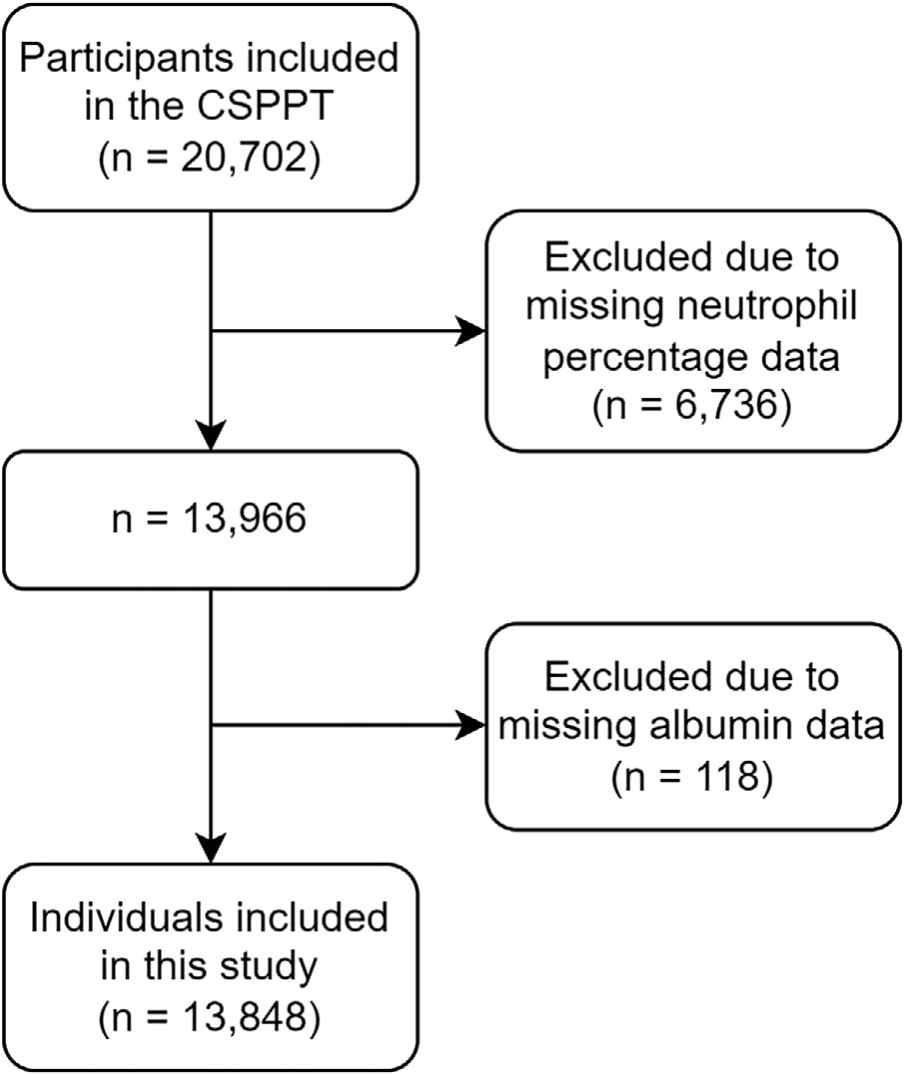
Flowchart showing the study inclusion and exclusion criteria: a cohort study based on the CSPPT. CSPPT, China Stroke Primary Prevention Trial.

This study was a post hoc analysis of the CSPPT data that aimed to determine the association between NPAR and the risk of first stroke. Overall, 20 702 subjects were included in the CSPPT, of whom 13 848 completed the final visit and had baseline neutrophil percentage and serum albumin level data available (see Figure 1).

### 2.2 Data Availability and Patient Consent

This research adhered to the Strengthening the Reporting of Observational Studies in Epidemiology (STROBE) guideline [31]. Both the parent study (CSPPT) and the present study were approved by the Ethics Committee of the Institute of Biomedicine, Anhui Medical University, Hefei, China (FWA assurance number FWA00001263). Written informed consent was obtained from all participants prior to the collection of their data.

## 3 Data Collection

### 3.1 Anthropometric Measurements

Anthropometric measurements, including height, weight, waist circumference, and hip circumference, were obtained for each participant. The body mass index (BMI) was calculated by dividing weight in kilograms by the square of height in meters. Systolic blood pressure (SBP) and diastolic blood pressure (DBP) were recorded using an electronic sphygmomanometer with the subject in a seated position following a 5‐min rest period.

### 3.2 Questionnaire

Detailed information on demographics, occupational background, medical history, medication usage, and lifestyle factors, including smoking status and alcohol consumption, was collected from all participants using a standardized questionnaire.

### 3.3 Laboratory Assays

Venous blood samples were collected after an overnight fast and transported to the laboratory within 30 min. The samples were analyzed to obtain a complete blood count and a biochemical profile. The biochemical profile included fasting lipid parameters (serum total cholesterol [TC], high‐density lipoprotein cholesterol [HDL‐C], and triglycerides [TG]), fasting blood glucose (FBG), plasma creatinine, and serum homocysteine, all of which were measured using automated analyzers (Beckman Coulter, Brea, CA) at the core laboratory of the National Clinical Research Center for Kidney Disease (Nanfang Hospital, Guangzhou, China). Estimated glomerular filtration rate (eGFR) was calculated using the Chronic Kidney Disease Epidemiology Collaboration (CKD‐EPI) method [32]. The neutrophil percentage was obtained from the complete blood count and represents the proportion of neutrophils among total white blood cells. The NPAR was calculated by dividing the neutrophil percentage by the albumin concentration using the following formula: NPAR = (neutrophil percentage [%] × 100)/albumin (g/dL).

## 4 Assessment of Outcomes

The primary outcome was the occurrence of first stroke, either nonfatal or fatal, with the exclusion of subarachnoid hemorrhage and silent stroke. All outcomes were independently reviewed and adjudicated by an Endpoint Adjudication Committee using established standard criteria.

## 5 Covariables

The covariates included in all analyses were age, sex, study center, treatment group, BMI, SBP, DBP, blood pressure on treatment, total homocysteine, fasting glucose, total cholesterol, eGFR, and smoking status.

## 6 Statistical Analysis

The data were analyzed using EmpowerStats (http://www.empowerstats.com) and R statistical software (version 3.2.3). NPAR was evaluated as a categorical variable, divided into quartiles (Q1–Q4), and compared both across quartiles and between Q1 and Q2–Q4. Differences in cumulative risk of stroke were compared across the different NPAR quartiles using Kaplan–Meier curve analysis. Continuous variables are expressed as the mean ± standard deviation (SD) or as the median (interquartile range), and categorical variables are reported as the count and percentage. Multivariable Cox proportional hazards regression models were used to estimate hazard ratios (HRs) and calculate 95% confidence intervals (CIs) for the association between the NPAR and the risk of stroke. The crude model was unadjusted, while the adjusted model accounted for age, sex, study center, treatment group, BMI, SBP, DBP, total homocysteine, FBG, TC, eGFR, and smoking status.

Potential modifications in the association between the NPAR and risk of stroke were explored in additional stratified analyses that included age (< 60 years [median] vs. ≥ 60 years), sex (male vs. female), BMI (< 25 vs. ≥25), SBP (< 164.7 mmHg [median] vs. ≥ 164.7 mmHg), DBP (< 94.0 mmHg [median] vs. ≥ 94.0 mmHg), eGFR (< 96.2 mL/min/1.73 m^2^ [median] vs. ≥ 96.2 mL/min/1.73 m^2^), FBG (< 5.5 mmol/L [median] vs. ≥ 5.5 mmol/L), homocysteine (< 12.5 µmol/L [median] vs. ≥ 12.5 µmol/L), TC (< 5.4 mmol/L [median] vs. ≥ 5.4 mmol/L), and smoking status (never, former, or current smoker). Interaction effects were analyzed by including multiplicative interaction terms in multivariable Cox proportional hazards models. A two‐sided *p* value of < 0.05 was considered statistically significant.

## 7 Results

### 7.1 Characteristics of the Study Participants

The baseline characteristics of the study participants are presented in Table 1. The study included 13,848 individuals with a mean age of 59.8 ± 7.6 years; 5572 (40.2%) were male and 8,276 were female. The mean age was 60.6 ± 7.7 years in men and 59.2 ± 7.4 years in women. The men were significantly older and had higher DBP and homocysteine levels but had lower BMI, SBP, FBG, TC, and eGFR. Men were also more likely to be former or current smokers.

**TABLE 1:** Baseline characteristics of the study participants.

Variables	Total	Male	Female	*p* value
***N***	13 848	5572	8276	
**Age, mean ± SD, years**	59.8 (7.6)	60.6 (7.7)	59.2 (7.4)	< 0.001
**BMI, mean ± SD, kg/m^2^**	25.2 (3.7)	24.5 (3.4)	25.7 (3.8)	< 0.001
**SBP, mean ± SD, mmHg**	167.9 (20.7)	166.2 (20.6)	169.1 (20.6)	< 0.001
**DBP, mean ± SD, mmHg**	94.7 (11.8)	96.3 (12.2)	93.7 (11.4)	< 0.001
**Laboratory results**				
FBG, median (IQR), mmol/L	5.5 (5.1–6.2)	5.5 (5.0–6.1)	5.6 (5.1–6.2)	< 0.001
TC, median (IQR), mmol/L	5.4 (4.8–6.2)	5.3 (4.6–6.1)	5.5 (4.9–6.3)	< 0.001
eGFR, median (IQR), mL/min per 1.73 m^2^	96.2 (88.3–102.5)	94.7 (86.8–101.5)	97.2 (89.5–103.1)	< 0.001
Homocysteine, median (IQR), µmol/L	12.5 (10.4–15.6)	14.1 (11.9–17.8)	11.5 (9.7–14.0)	< 0.001
**Smoking status**				< 0.001
Never	9662 (69.8)	1762 (31.6)	7900 (95.5)	
Former	1075 (7.8)	957 (17.2)	118 (1.4)	
Current	3107 (22.4)	2853 (51.2)	254 (3.1)	
**NPAR, median (IQR)**	12.0 (10.7–13.4)	12.3 (11.0–13.7)	11.8 (10.5–13.2)	< 0.001

*Note*: The data are presented as the mean (SD), median (IQR), or number (percentage). A *p* value < 0.05 indicates a significant difference between or across groups.

Abbreviations: BMI, body mass index; DBP, diastolic blood pressure; eGFR, estimated glomerular filtration rate; FBG, fasting blood glucose; IQR, interquartile range; NPAR, neutrophil percentage‐to‐albumin ratio; SBP, systolic blood pressure; SD, standard deviation; TC, total cholesterol.

### 7.2 Association Between NPAR and Stroke

**FIGURE 2:**
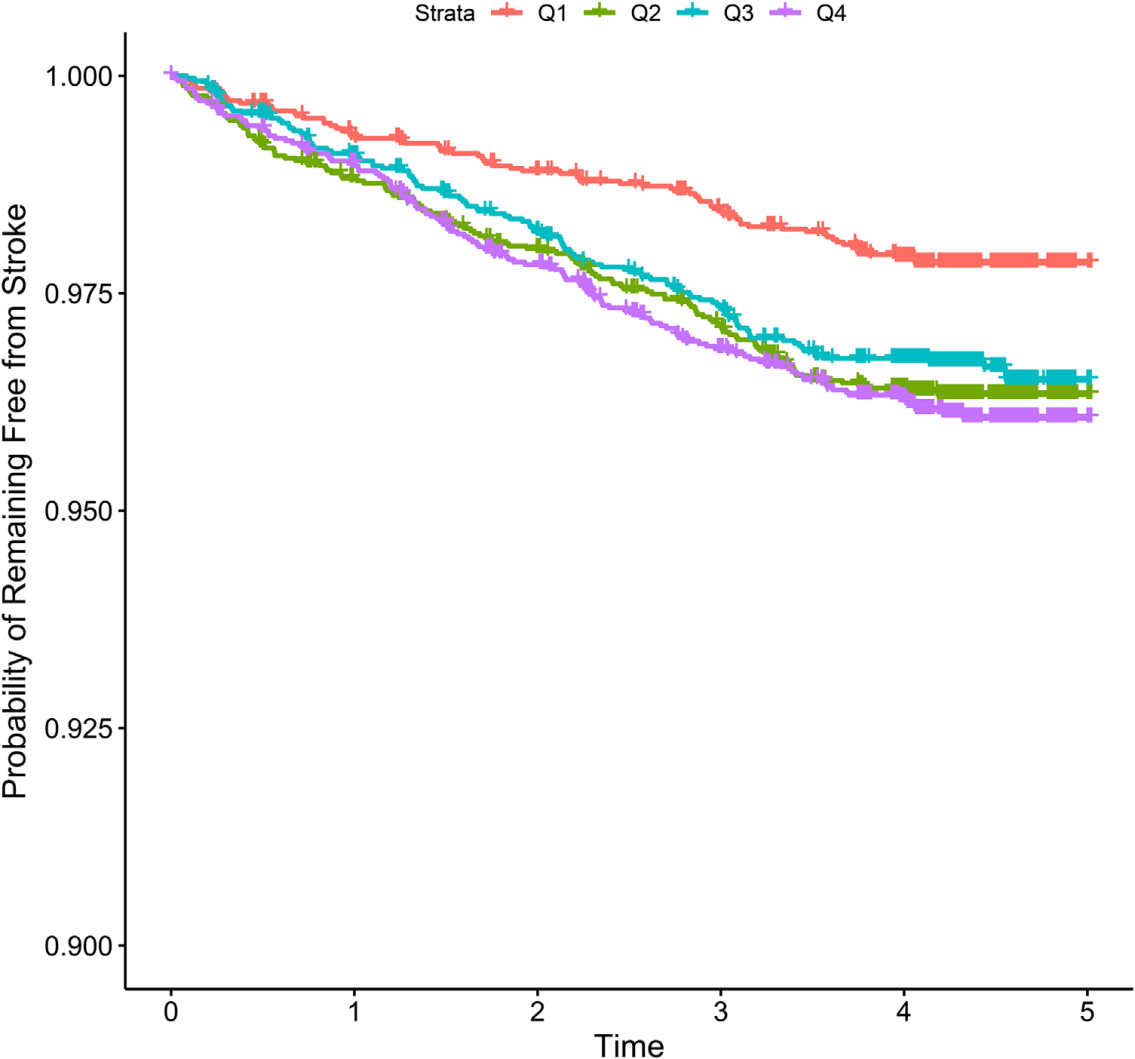
Kaplan–Meier estimates for stroke events. The panel presents the Kaplan–Meier curves for stroke in 13 848 subjects divided into four quartiles according to their NPAR. NPAR, neutrophil percentage‐to‐albumin ratio.

During a median follow‐up of 4.5 years, 371 subjects (2.7%) experienced a stroke event. The time‐to‐event analysis is shown in Figure 2. The Kaplan–Meier curve for cumulative incidence of stroke stratified by quartile of baseline NPAR demonstrated that the probability of not experiencing a stroke event was significantly higher in the Q1 group than in the Q2, Q3, and Q4 groups.

Table 2 presents the results of the Cox regression analyses examining the association between NPAR and the risk of stroke. Participants were divided into quartiles based on their NPAR for analysis. In the total population, the risk of stroke was significantly higher in the Q2, Q3, and Q4 groups (HR 1.72, 95% CI 1.29–2.29, *p* < 0.001 [Q2], HR 1.55, 95% CI 1.16–2.08, *p* = 0.003 [Q3], and HR 1.78, 95% CI 1.34–2.37, *p* < 0.001 [Q4]) than in the Q1 group (NPAR < 10.7) in the crude model. This association persisted after adjustment for demographic and other confounding factors (HR 1.76, 95% CI 1.31–2.36, *p* < 0.001 [Q2], HR 1.54, 95% CI 1.14–2.08, *p* = 0.005 [Q3], and HR 1.57, 95% CI 1.17–2.12, *p* = 0.003 [Q4]). The increased risk of stroke remained consistent when subjects in the Q2, Q3, and Q4 groups were combined and compared with those in the Q1 group in both the crude model (HR 1.68, 95% CI 1.31–2.16) and the adjusted model (HR 1.62, 95% CI 1.25–2.10).

**TABLE 2:** Association between baseline NPAR and the risk of stroke.

NPAR	*N*	Events (%)	Crude model		Adjusted model^a^	
			HR (95%CI)	*p* value	HR (95%CI)	*p* value
**Total**						
Quartiles						
Q1 (< 10.7)	3461	74 (2.1)	Ref		Ref	
Q2 (10.7–12.0)	3463	126 (3.6)	1.72 (1.29, 2.29)	< 0.001	1.76 (1.31, 2.36)	< 0.001
Q3 (12.0–13.4)	3462	114 (3.3)	1.55 (1.16, 2.08)	0.003	1.54 (1.14, 2.08)	0.005
Q4 (≥ 13.4)	3462	130 (3.8)	1.78 (1.34, 2.37)	< 0.001	1.57 (1.17, 2.12)	0.003
Categories						
Q1 (< 10.7)	3461	200 (2.9)	Ref		Ref	
Q2–Q4 (≥ 10.7)	10 387	244 (3.5)	1.68 (1.31, 2.16)	< 0.001	1.62 (1.25, 2.10)	< 0.001
**Male**						
Quartiles						
Q1 (< 10.7)	1114	38 (3.4)	Ref		Ref	
Q2 (10.7–12.0)	1330	52 (3.9)	1.15 (0.76, 1.75)	0.514	1.21 (0.79, 1.87)	0.381
Q3 (12.0–13.4)	1453	55 (3.8)	1.11 (0.73, 1.68)	0.623	1.12 (0.73, 1.73)	0.598
Q4 (≥ 13.4)	1675	68 (4.1)	1.20 (0.81, 1.79)	0.368	1.16 (0.76, 1.76)	0.492
Categories						
Q1 (< 10.7)	1114	38 (3.4)	Ref		Ref	
Q2–Q4 (≥ 10.7)	4458	175 (3.9)	1.16 (0.81, 1.64)	0.420	1.16 (0.80, 1.68)	0.422
**Female**						
Quartile						
Q1 (< 10.7)	2347	36 (1.5)	Ref		Ref	
Q2 (10.7–12.0)	2133	74 (3.5)	2.29 (1.54, 3.41)	< 0.001	2.36 (1.57, 3.53)	< 0.001
Q3 (12.0–13.4)	2009	59 (2.9)	1.93 (1.28, 2.93)	0.002	2.01 (1.32, 3.07)	0.001
Q4 (≥ 13.4)	1787	62 (3.5)	2.29 (1.52, 3.46)	< 0.001	2.00 (1.31, 3.06)	< 0.001
Categories						
Q1 (< 10.7)	2347	36 (1.5)	Ref		ref	
Q2–Q4 (≥ 10.7)	5929	195 (3.3)	2.17 (1.52, 3.10)	< 0.001	2.13 (1.48, 3.07)	< 0.001

*Note*: A *p* value < 0.05 indicates a significant difference between or across groups.

Abbreviations: CI, confidence interval; HR, hazard ratio; NPAR, neutrophil percentage‐to‐albumin ratio.

^a^ Adjusted for age, sex, center, treatment group, body mass index, systolic and diastolic blood pressure, total homocysteine, fasting blood glucose, total cholesterol, estimated glomerular filtration rate, smoking status, and blood pressure on treatment.

Consistent with the findings for the total population, the risk of stroke was significantly higher in women with an elevated NPAR. In women with hypertension, the risk of stroke was increased in both the crude model (HR 2.29, 95% CI 1.54‐3.41, *p* < 0.001 [Q2], HR 1.93, 95% CI 1.28–2.93, *p* = 0.002 [Q3], and HR 2.29, 95% CI 1.52–3.46, *p* < 0.001 [Q4]) and the adjusted model (HR 2.36, 95% CI 1.57–3.53, *p* < 0.001 [Q2], HR 2.01, 95% CI 1.32–3.07, *p* = 0.001 [Q3], and HR 2.00, 95% CI 1.31–3.06, *p* < 0.001 [Q4]). For participants with an NPAR of ≥ 10.7, the HR for stroke was 2.17 (95% CI 1.52–3.10) in the crude model and 2.13 (95% CI 1.48–3.07) in the adjusted model.

The association between the NPAR and stroke did not reach statistical significance in men. Compared with male participants in the Q1 group, those in the Q2, Q3, and Q4 groups did not show an increased risk of stroke in either the crude model or adjusted model (*p* > 0.05). The results remained unchanged when male participants in the Q2, Q3, and Q4 groups were combined and compared with those in the Q1 group.

### 7.3 Assessment of Interaction

Table 3 shows the results of the modification effects of NPAR on the risk of stroke across various subgroups. The association between NPAR and incidence of stroke was notably stronger in women than in men (HR 2.13 vs. 1.16, *p* for interaction 0.035). No significant modification effect of age, BMI, SBP, DBP, eGFR, FBG, homocysteine, TC, or smoking status was observed (*p* for interaction, all > 0.05).

**TABLE 3:** Subgroup analyses of the association between the NPAR and the risk of stroke.^a^

Subgroup	NPAR Q1 (< 10.7)		NPAR Q2–Q4 (≥ 10.7)		HR (95% CI)	*p* for Interaction
	*N*	Events (%)	*N*	Events (%)		
**Age, years**						0.828
< 60 (median)	1940	32 (1.6)	4984	126 (2.5)	1.64 (1.09, 2.48)	
≥ 60	1521	42 (2.8)	5403	244 (4.5)	1.70 (1.22, 2.38)	
**Sex**						0.035
Male	1114	38 (3.4)	4458	175 (3.9)	1.16 (0.80, 1.68)	
Female	2347	36 (1.5)	5929	195 (3.3)	2.13 (1.48, 3.07)	
**BMI, kg/m^2^**						0.594
< 25	1589	29 (1.8)	5327	172 (3.2)	1.75 (1.16, 2.64)	
≥ 25	1871	45 (2.4)	5058	198 (3.9)	1.52 (1.09, 2.12)	
**SBP, mmHg**						0.349
< 164.7 (Median)	1781	28 (1.6)	5083	112 (2.2)	1.32 (0.86, 2.03)	
≥ 164.7	1680	46 (2.7)	5304	258 (4.9)	1.77 (1.28, 2.45)	
**DBP, mmHg**						0.386
< 94.0 (Median)	1756	35 (2.0)	5139	156 (3.0)	1.42 (0.97, 2.06)	
≥ 94.0	1705	39 (2.3)	5248	214 (4.1)	1.82 (1.27, 2.61)	
**eGFR**						0.490
< 96.2 (Median)	1540	39(2.5)	5274	223(4.2)	1.47 (1.04, 2.07)	
≥ 96.2	1866	31 (1.7)	4949	135 (2.7)	1.85 (1.24, 2.76)	
**Fasting glucose, mmol/L**						0.355
< 5.5 (Median)	1630	33 (2.0)	5174	158 (3.1)	1.37 (0.93, 2.00)	
≥ 5.5	1776	37 (2.1)	5050	200 (4.0)	1.82 (1.28, 2.60)	
**Homocysteine, µmol/L**						0.252
< 12.5 (Median)	1892	29 (1.5)	4966	147 (3.0)	1.96 (1.31, 2.95)	
≥ 12.5	1531	42 (2.7)	5338	215 (4.0)	1.39 (0.99, 1.94)	
**Total cholesterol, mmol/L**						0.269
< 5.4 (Median)	1559	22 (1.4)	5205	149 (2.9)	1.90 (1.21, 2.99)	
≥ 5.4	1847	48 (2.6)	5020	209 (4.2)	1.47 (1.07, 2.01)	
**Smoking status**						0.449
Never	2634	44 (1.7)	7028	222 (3.2)	1.85 (1.32, 2.58)	
Former	243	9 (3.7)	832	39 (4.7)	1.27 (0.58, 2.79)	
Current	584	21 (3.6)	2523	109 (4.3)	1.23 (0.75, 2.00)	

*Note*: A *p* value < 0.05 indicates a significant difference between or across groups.

Abbreviations: BMI, body mass index; CI, confidence interval; DBP, diastolic blood pressure; eGFR, estimated glomerular filtration rate; FBG, fasting blood glucose; HR, hazard ratio; NPAR, neutrophil percentage‐to‐albumin ratio; SBP, systolic blood pressure; TC, total cholesterol.

^a^ Adjusted, if not stratified, for age, sex, center, treatment group, BMI, SBP, DBP, total homocysteine, TC, FBG, eGFR, smoking status, and blood pressure on treatment.

## 8 Discussion

This large‐scale cohort study found an association between a higher NPAR and an elevated risk of stroke independent of traditional cardiovascular risk factors in a Chinese population with hypertension. Importantly, our results revealed a pronounced sex‐specific difference: the association between NPAR and stroke was significant in women but not in men.

Recent studies have highlighted NPAR as a biomarker of systemic inflammatory activity that demonstrates a positive correlation with traditional inflammatory markers such as C‐reactive protein (CRP) [15]. Investigations into the relationships between NPAR and various clinical outcomes, including complications from cardiovascular, cerebrovascular, renal, and infectious diseases, have yielded promising results [9, 10, 11, 12, 13, 14, 15, 16, 17, 33, 34] Studies by Xu et al. and Yu et al. found that the NPAR has strong predictive performance in terms of all‐cause and cardiovascular mortality in patients undergoing peritoneal dialysis, outperforming traditional markers like CRP and novel inflammatory biomarkers such as the platelet‐lymphocyte ratio [22, 23]. Furthermore, Zhang et al. found that NPAR had superior predictive ability for stroke‐associated infections when compared with albumin or the neutrophil percentage alone [25]. Despite these findings, the potential of NPAR to predict the incidence of stroke in patients with hypertension has not been explored.

This study demonstrated a significant correlation between NPAR and risk of stroke in patients with hypertension. The underlying mechanisms may be linked to the associations between an elevated neutrophil percentage, a reduced albumin level, and inflammation. The neutrophil percentage is a well‐established marker of inflammation [35], and a low serum albumin level (i.e., hypoalbuminemia) is also associated with the severity of inflammation and a heightened susceptibility to infectious complications. Inflammatory processes increase capillary permeability, leading to escape of serum albumin into the interstitial space, expansion of the interstitial volume, and altered distribution of albumin [36]. Lan et al. also reported that elevated neutrophil levels were associated with increased all‐cause mortality and mortality from chronic lower respiratory disease (HR 1.13 and 1.34, respectively), whereas higher albumin levels were associated with reduced all‐cause and cardiovascular mortality (HR 0.91 and 0.86) [37].

All the common causes of stroke, including atherosclerosis, hypertension, diabetes, and infection, contribute to a pro‐inflammatory systemic environment [38, 39]. A substantial body of evidence indicates that inflammation plays a critical role throughout the stages of development of atherosclerosis. It contributes significantly to atherosclerosis, thrombosis, and cerebral small vessel disease, thereby increasing the risk of both ischemic and hemorrhagic stroke [7, 40, 41]. Inflammatory biomarkers, including interleukins, CRP, and vascular cell adhesion molecule 1, have been demonstrated to have value in the early diagnosis of ischemic stroke and in predicting clinical outcomes [42].

Although the risk of stroke was significantly elevated in participants with NPAR levels in Q2–Q4 compared with those in Q1, the hazard ratios across the upper three quartiles did not demonstrate a progressively increasing trend. The absence of a progressively increasing trend across Q2–Q4 suggests that the association between NPAR and stroke risk follows a threshold‐type pattern rather than a linear dose‐response relationship. Once NPAR exceeds approximately 10.7, the transition from Q1 to Q2, the systemic inflammatory burden may reach a biologically relevant threshold sufficient to activate pro‐atherogenic and pro‐thrombotic pathways, thereby sharply elevating stroke risk. Beyond this threshold, further increases in NPAR contribute minimally to additional risk, resulting in a plateau phenomenon. This nonlinear behavior is consistent with the known saturation characteristics of inflammatory biomarkers, in which risk rises rapidly when inflammation first becomes elevated but subsequently stabilizes after inflammatory pathways are fully activated [43, 44].

In the present study, we observed that the correlation between NPAR and the incidence of stroke was more pronounced in women, suggesting that NPAR may be a stronger predictor of stroke in female patients with hypertension than in their male counterparts.

The sex differences in stroke epidemiology are likely attributable to both socioeconomic and biological factors. A possible explanation may lie in the fact that men are more likely to have traditional risk factors for stroke, such as smoking, dyslipidemia, and obesity, which contribute significantly to their heightened risk of stroke. Therefore, it can be inferred that the strong predictive power of these conventional risk factors may diminish the contribution of non‐traditional risk factors (e.g., NPAR) and the role of inflammation in the pathogenesis of stroke in men [45, 46]. By contrast, women generally have fewer traditional risk factors, which may accentuate the role of inflammation in the pathogenesis of stroke in the female population [47].

The present study is the first to investigate the association between NPAR and risk of stroke in individuals with hypertension while considering a range of potential confounding factors. The potential role of NPAR in stroke primary prevention suggests several avenues for future research. Larger prospective studies are needed to validate our findings in broader populations and to explore whether longitudinal changes in NPAR improve risk stratification. Moreover, the incremental value of NPAR when added to existing stroke risk models is yet to be explored to further clarify its clinical utility. Nevertheless, it has some limitations. First, the laboratory test results required for calculation of NPAR were obtained from a single baseline measurement, which may not accurately represent inflammatory status in the long term. Second, covariates collected at baseline may change over time, and residual or unmeasured confounding factors cannot be entirely ruled out. Finally, owing to the observational nature of the study, our findings cannot be used to infer causality.

## 9 Conclusions

In this nationwide hypertensive cohort, a higher NPAR was significantly associated with an increased risk of first stroke, independent of traditional cardiovascular risk factors. Notably, the association was substantially stronger in women, indicating that inflammation‐related mechanisms may contribute disproportionately to stroke pathogenesis in female patients with hypertension. These findings underscore the need for sex‐specific risk assessment and preventive strategies, and position NPAR as a promising, easily obtainable biomarker for identifying women at elevated cerebrovascular risk.

## Author Contributions

L.W. and Z.L. contributed to the conception of the work, L.W. and N.Z. contributed to the design of the work, Z.L. and N.Z. contributed to data analysis, X.L., L.Z., and F.F. contributed to data interpretation, and L.W. and Z.L. managed the presentation of the manuscript. J.L. and Y.Z. were responsible for supervision and revision of the work. All authors have approved the submitted version and agreed both to be personally accountable for their own contributions and to ensure that questions related to the accuracy or integrity of any part of the work, even ones in which the author was not personally involved, are appropriately investigated and resolved and that the resolution is documented in the literature.

## Funding

This work was supported by the Noncommunicable Chronic Diseases–National Science and Technology Major Project (grant Nos. 2023ZD0503400 and 2023ZD0503402); the National Natural Science Foundation of China (grant No. 82370442); and the Capital’s Funds for Health Improvement and Research (grant No. 2024‐1‐4071).

## Ethics Statement

Both the parent study (CSPPT) and the present study were approved by the Ethics Committee of the Institute of Biomedicine, Anhui Medical University, Hefei, China (FWA assurance number FWA00001263). Written informed consent was obtained from all participants before data collection.

## Consent

The authors have nothing to report.

## Conflicts of Interest

The authors declare no conflicts of interest and there has been no financial relationship with commercial entities to be disclosed. All authors completed the ICMJE (International Committee of Medical Journal Editors) conflict of interest form.

## Data Availability

The datasets utilized for analysis in the current study are available from the corresponding author upon reasonable request.
